# Identification of the shared gene signatures and pathways between polycystic ovary syndrome and endometrial cancer: An omics data based combined approach

**DOI:** 10.1371/journal.pone.0271380

**Published:** 2022-07-13

**Authors:** Chenyun Miao, Yun Chen, Xiaojie Fang, Ying Zhao, Ruye Wang, Qin Zhang

**Affiliations:** 1 Department of TCM Gynecology, Hangzhou TCM Hospital Affiliated to Zhejiang Chinese Medical University, Hangzhou, Zhejiang, China; 2 Department of Anorectal Surgery, Hangzhou TCM Hospital Affiliated to Zhejiang Chinese Medical University, Hangzhou, Zhejiang, China; Jamia Millia Islamia, INDIA

## Abstract

**Objective:**

Polycystic ovary syndrome (PCOS) is a common endocrine disorder with high incidence. Recently it has been implicated as a significant risk factor for endometrial cancer (EC). Our study aims to detect shared gene signatures and biological mechanism between PCOS and EC by bioinformatics analysis.

**Methods:**

Bioinformatics analysis based on GEO database consisted of data integration, network construction and functional enrichment analysis was applied. In addition, the pharmacological methodology and molecular docking was also performed.

**Results:**

Totally 10 hub common genes, MRPL16, MRPL22, MRPS11, RPL26L1, ESR1, JUN, UBE2I, MRPL17, RPL37A, GTF2H3, were considered as shared gene signatures for EC and PCOS. The GO and KEGG pathway analysis of these hub genes showed that “mitochondrial translational elongation”, “ribosomal subunit”, “structural constituent of ribosome” and “ribosome” were highly correlated. Besides, associated transcription factors (TFs) and miRNAs network were constructed. We identified candidate drug molecules including fenofibrate, cinnarizine, propanil, fenthion, clindamycin, chloramphenicol, demeclocycline, hydrochloride, azacitidine, chrysene and artenimol according to these hub genes. Molecular docking analysis verified a good binding interaction of fenofibrate against available targets (JUN, ESR1, UBE2I).

**Conclusion:**

Gene signatures and regulatory biological pathways were identified through bioinformatics analysis. Moreover, the molecular mechanisms of these signatures were explored and potential drug molecules associated with PCOS and EC were screened out.

## Introduction

Polycystic ovary syndrome (PCOS) is characterized by high incidence rate of 5–12% [1, 2] and is one of the most frequently occurring endocrine disorders in women of reproductive age. Characteristics of PCOS include oligo/anovulation, hyperandrogenism and polycystic ovaries, and is associated with heterogeneous clinical presentations such as menstrual irregularity, infertility, hirsutism and insulin resistance [3]. Aided by advances in research that help understand the biological processes implicated in PCOS, it also has been confirmed to have links to cancers in the endometrium, ovaries, kidneys, hematopoietic and pancreas system [4].

Endometrial cancer (EC) is the most common gynecologic cancer in the Western world with rising incidence and mortality [5]. It is estimated to lead to around 76000 deaths worldwide annually [6]. Published meta-analyses report that PCOS is a significant risk factor for EC [7], the results of which show that women with PCOS have 3-fold higher risk of developing EC compared with women without PCOS [7, 8]. Women aged less than 54 years have a significantly high risk for EC compared with elderly women (OR, 4.05) [9]. Features for PCOS such as obesity and anovulation can increase estrogen level and progesterone resistance, leading to development of endometrial hyperplasia and ultimately EC [10, 11]. Intricate relationship between EC and PCOS has been recognized for a number of years, but the exact pathomechanism mainly the genetic relationship between PCOS and EC remains unclear.

Gene expression profiles analysis and bioinformatic analysis using microarray data have been widely used to identify characteristic patterns of gene expression, dysregulated biological pathways, and gene interactome. In the current study, we utilized a range of bioinformatic approach to screen common genes and to explore transcriptional regulatory networks consist of transcription factors (TFs) and miRNAs between PCOS and EC to identify common molecular signatures and potential mechanisms. Finally, potential drug molecules were suggested. This study can help understand the molecular mechanism of this association and provide information for therapeutic strategy of PCOS patients with EC, which is of some clinical implications.

## Material and methods

### Retrieval of gene expression data

Microarray data were retrieved from the Gene Expression Omnibus (GEO) (http://www.ncbi.nih.gov/geo) [12]. GSE48301 dataset analyzed using Agilent GPL6244 platform [HuGene-1_0-st] comprised whole genome expression arrays of different endometrial cell populations obtained from PCOS women (n = 6) and healthy controls (n = 6). GSE115810 dataset analyzed using Agilent GPL96 platform [HG-U133A] comparing gene expression arrays from normal human endometrium (n = 3) with gene expression arrays from endometrial cancer of different grades (n = 24).

### Identification of DEGs and shared gene signatures between EC and PCOS

GEO2R (https://www.ncbi.nlm.nih.gov/geo/geo2r/) is a web-based analysis tool that uses GeoQuery and Limma R packages for data analysis [12]. Differentially expressed genes (DEGs) were analyzed using a p value < 0.05 as the cut-off criteria. Common DEGs between GSE48301 and GSE115810 datasets, which were potential genes associated with EC risk in women with PCOS were identified by R software (version 4.0.3) and visualized by the Venn diagram (http://bioinformatics.psb.ugent.be/webtools/Venn/).

### Protein-protein interaction (PPI) network analysis and identification of hub targets

A PPI network was constructed using STRING tool (https://string-db.org/) to further explore the interaction between the overlapping DEGs [13]. All interaction evidence contributes to nodes in a given network is scored, resulting in an interaction score [14]. The minimum interaction score was set as greater than 0.4, and unconnected nodes in the network were removed. Further, key nodes within the PPI network were selected as hub genes using cytohubba plugin in Cytoscape software [15]. Hub genes were selected mainly based on their Maximal Clique Centrality (MCC) algorithm, which indicates essentiality of nodes in biological network [16]. Given a node *v*, the MCC of *v* is defined as MCC(*v*) = ∑_*C*∈S(*v*)_(|*C*|-1)!, where S(*v*) is the collection of maximal cliques which contain *v*, and (|*C*|-1)! is the product of all positive integers less than |*C*| [16].

### Functional enrichment analysis

Gene Ontology (GO) [17] and Kyoto Encyclopedia of Genes and Genomes (KEGG) enrichment analysis [18] of hub genes were performed using ClusterProfiler package in R (version 4.0.3) to determine the biological functions and signaling pathways associated with the hub genes. GO enrichment analysis is comprised of three main categories included biological process (BP), cell component (CC) and molecular function (MF). A statistical threshold criterion at a p-value < 0.05 was chosen for selecting significantly enriched GO terms and pathways.

### TFs-genes-miRNAs interaction network

Network analyst 3.0 tool (https://www.networkanalyst.ca/) is an online visual analytical platform for comprehensive gene expression profiling [19]. All hub genes were uploaded to network analyst to identify TFs and miRNAs that potentially regulated the hub genes. Genes-TFs network and genes-miRNAs network were also constructed using the cytohubba plugin in Cytoscape according to MCC score and degree.

### Identification of drug candidates and molecular docking

DSigDB database comprises of 19531 genes and 17389 compounds and provides a direct link between genes and drugs for drug development studies and translational research [20]. DSigDB database is accessed through Enrichr (https://amp.pharm.mssm.edu/Enrichr/) webserver and is used for analysis of the relationship between drugs and potential targets. Hub genes were uploaded to the database to find potential drug molecules for PCOS and EC that target these genes. The compounds were then sorted based on the adjusted p value (p<0.05) and the combined score that calculated using the p-value and z-score computed by assessing the deviation from the expected rank [21].

To explore potential binding of the drug candidates to hub genes, the 3D structures of the drug molecules were obtained from PubChem. In addition, crystal structures of target proteins were retrieved from the RCSB protein data bank (http://www.rcsb.org/) [22]. Molecular docking was then performed using AutoDock Vina tools, and the results were visualized using PyMol 2.4.0 [23, 24].

## Results

### DEGs and common genes were identified between PCOS and EC

**Fig 1:**
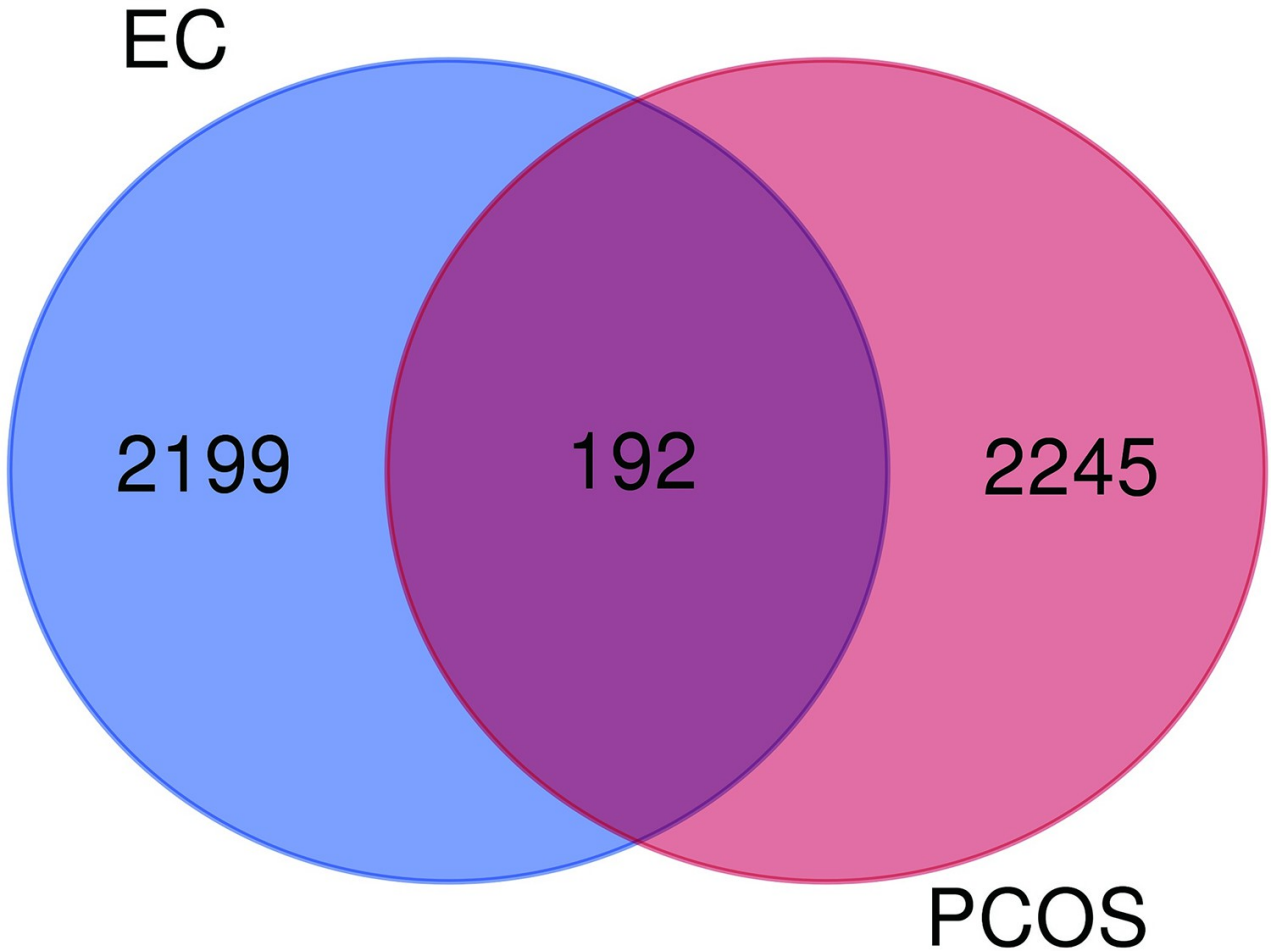
Venn diagram of the intersections of PCOS and EC. Intersections represent the differentially expressed genes in PCOS associated data series and EC associated data series.

GSE48301 dataset was used to explore DEGs for PCOS. The findings showed that a total of 2437 DEGs were identified from GSE48301 dataset. In addition, 2391 DEGs associated with EC were identified from GSE115810 dataset. Identification of overlapping genes between PCOS and EC was performed using R software. Visualization using the Venn diagram showed 192 common genes in PCOS and EC (Fig 1).

### Identification of hub genes

**Fig 2:**
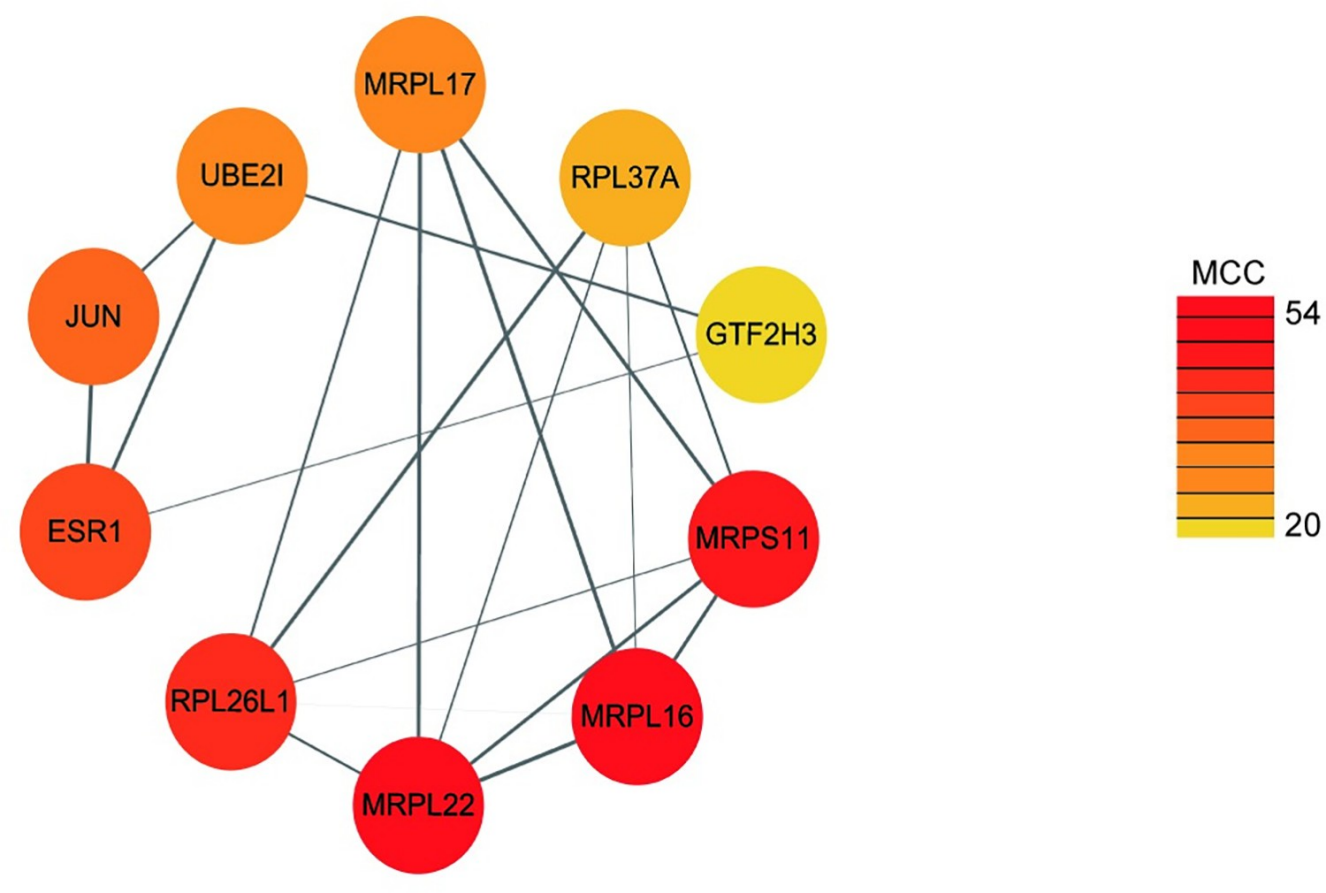
Hub genes screening. The color of the nodes are illustrated from red to yellow in descending order of MCC score. Gray lines highlight the interactions; line thickness refers to the interaction score provided by STRING.

The 192 common genes were submitted to STRING 11.0 database for construction of a medium confidence (score>0.4) PPI network. MCC score-based assessment was used to further identifythe hub genes using the cytohubba plugin. The top 10 genes including MRPL16, MRPL22, MRPS11, RPL26L1, ESR1, JUN, UBE2I, MRPL17, RPL37A, GTF2H3 were considered as hub genes (Table 1). The network comprised of 15 nodes and 30 edges (Fig 2).

**Table 1:** Hub genes identified through PPI analysis.

Hub genes	MCC	Description
MRPL16	54	Mitochondrial Ribosomal Protein L16
MRPL22	54	Mitochondrial Ribosomal Protein L22
MRPS11	50	Mitochondrial Ribosomal Protein S11
RPL26L1	49	Ribosomal Protein L26 Like 1
ESR1	42	Estrogen Receptor 1
JUN	40	Jun Proto-Oncogene, AP-1 Transcription Factor Subunit
UBE2I	34	Ubiquitin Conjugating Enzyme E2 I
MRPL17	34	Mitochondrial Ribosomal Protein L17
RPL37A	27	Ribosomal Protein L37a
GTF2H3	20	General Transcription Factor IIH Subunit 3

Maximal Clique Centrality (MCC) scores indicated essentiality of the gene in biological network. the greater the value, the more important the gene.

### Functional enrichment analysis

**Fig 3:**
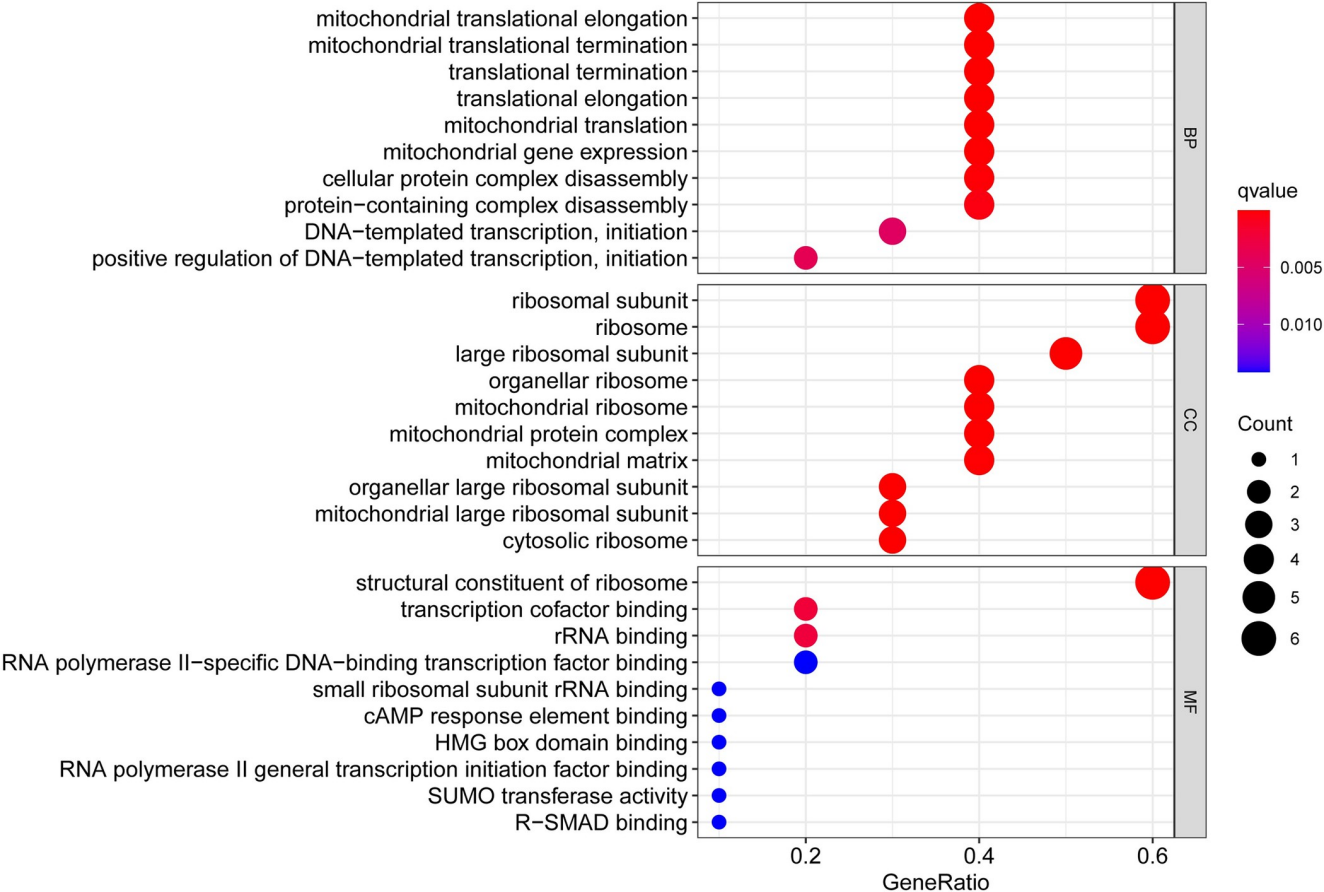
GO enrichment analysis. Shown are the top 10 most significantly enriched terms of each category based on p-value. The bubbles’ sizes are scaled according to the count of the potential targets enriched in the pathways. The bubbles are colored from red to blue in descending order of p-value. BP, biological process; CC, cell component; MF, molecular function.

**Fig 4:**
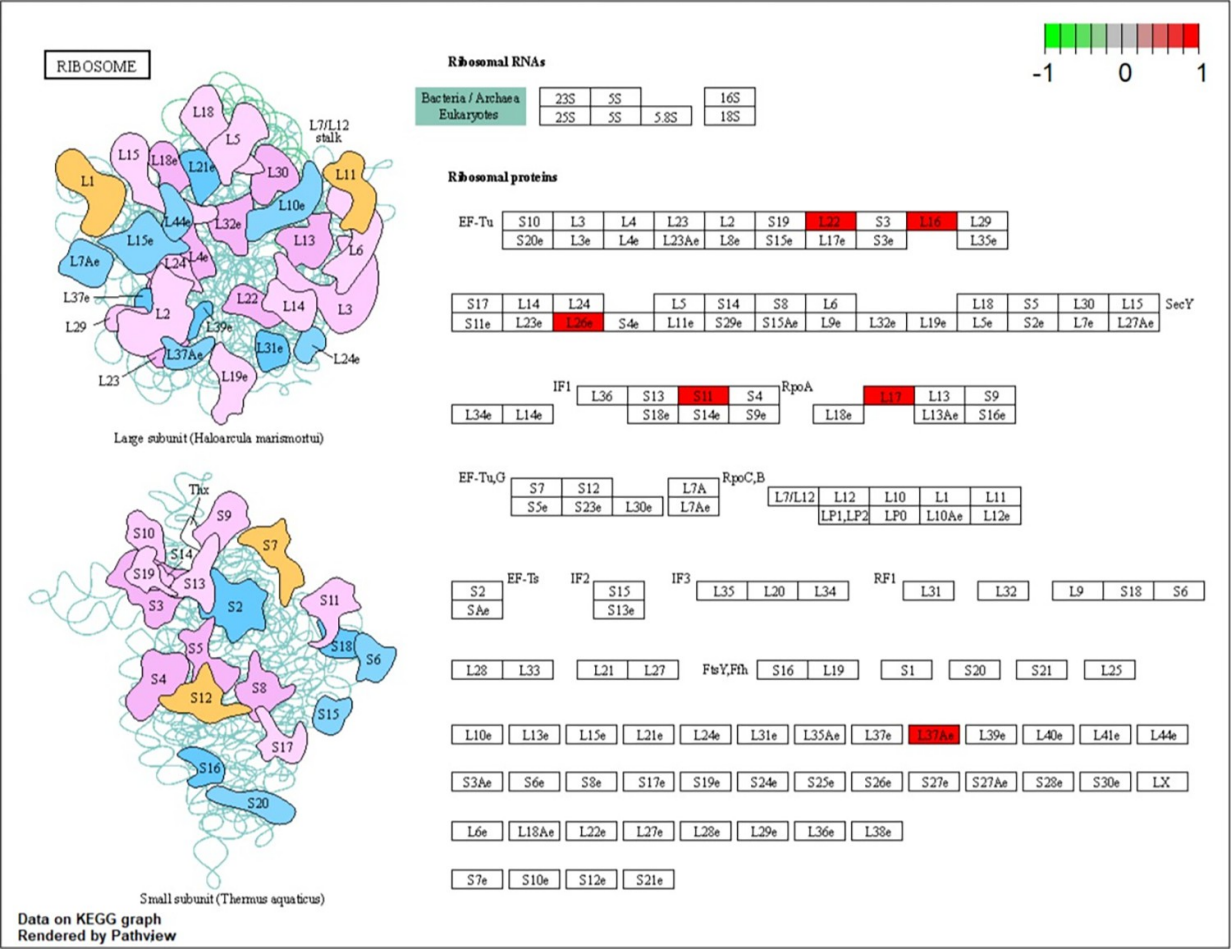
Selected KEGG pathways. Red nodes represent hub genes of PCOS and EC.

The findings indicated that several GO terms were enriched by the hub genes including 124 BP terms, 31 CC terms and 30 MF terms. Analysis of individual modules showed that “mitochondrial translational elongation”, “ribosomal subunit”, and “structural constituent of ribosome” were the most significantly enriched terms (Fig 3). KEGG pathway analysis was performed to identify dysregulated pathways enriched by the hub genes identified for PCOS and EC. The findings for KEGG pathways analysis showed that only one pathway, ribosome, was significantly enriched (Fig 4).

### Transcriptional signatures

**Fig 5:**
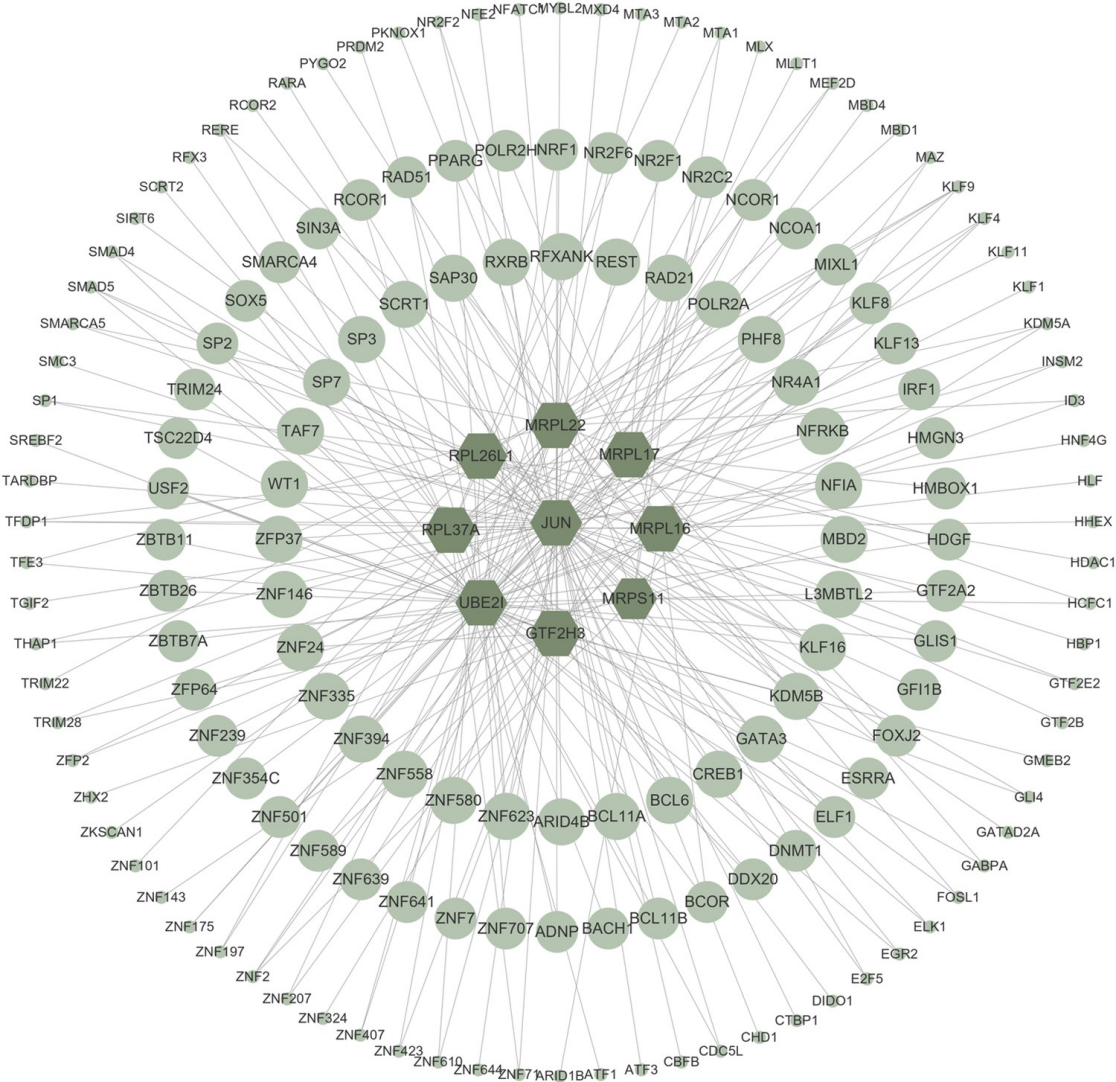
Genes-TFs interaction network. Hexagons represent hub genes; circle nodes represent TFs associated with hub genes.

**Fig 6:**
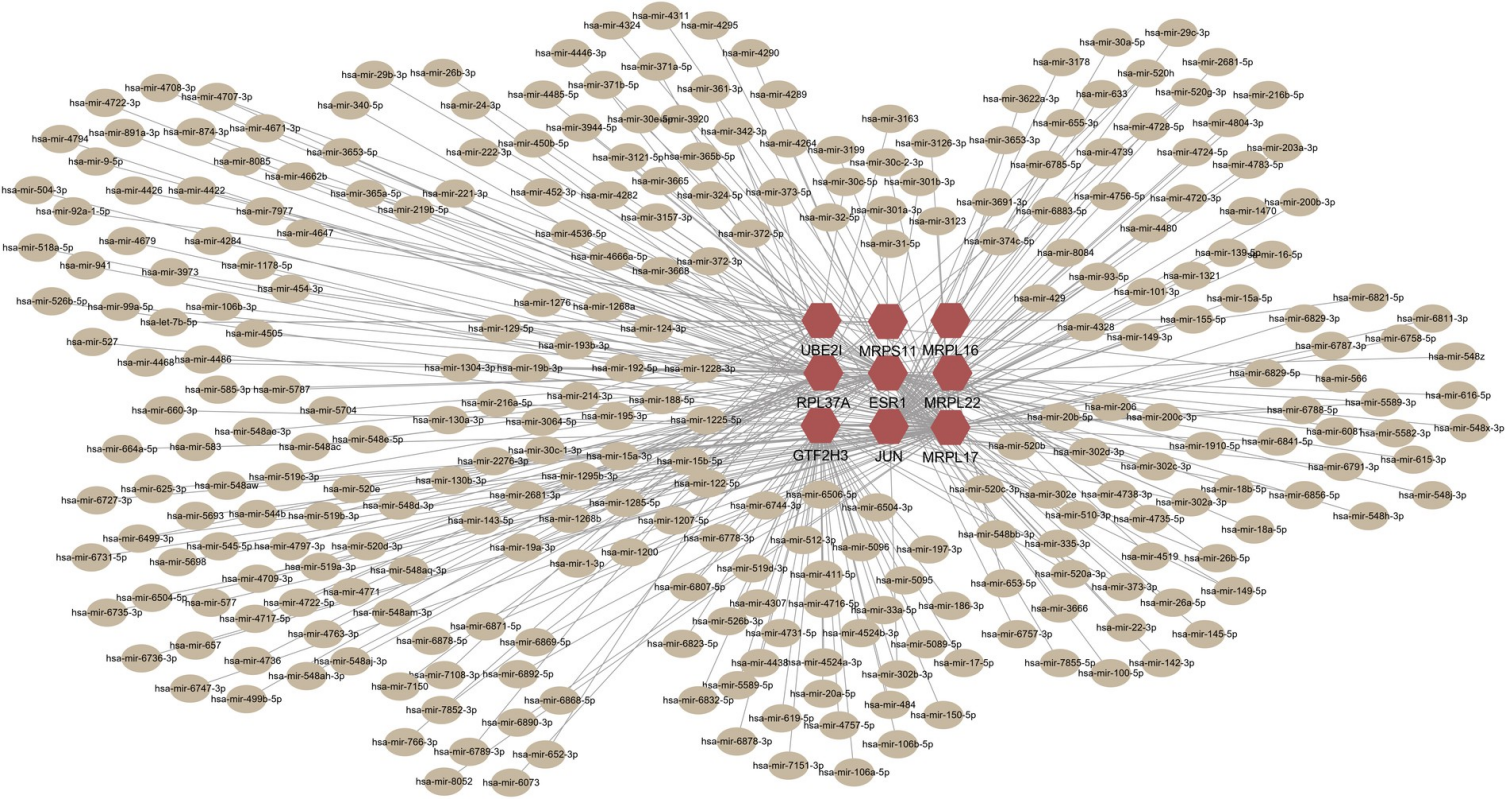
Genes-miRNAs interaction network. Hexagons represent hub genes; circle nodes represent miRNAs associated with hub genes.

Genes-TFs and genes-miRNAs interaction networks were reconstructed using experimentally verified interactions in the NetworkAnalyst platform to explore transcriptional signatures and post-transcriptional regulatory signatures [19]. There were 298 nodes and 316 edges in the genes-miRNAs network and 171 nodes and 244 edges in the genes-TFs network. Four TFs showed strong correlation with the hub genes namely, KLF9, PHF8, KDM5B, and SAP30(Fig 5). Nevertheless, no significantly correlated miRNAs were screened out in the cytohubba, MCC scores of all the related miRNAs were in the range of 1–2 (Fig 6).

### Candidate small drug molecules

The identified hub genes for PCOS and EC were uploaded to Enrichr platform. The platform provides a list of potential molecules that target the genes based on data from DSigDB database. The top ten candidate drug molecules were generated after manually removing duplicates based on the adjusted p-value. The drug molecules were fenofibrate, cinnarizine, propanil, fenthion, clindamycin, chloramphenicol, demeclocycline, hydrochloride, azacitidine, chrysene and artenimol (Table 2). Fenofibrate was with the highest combined score.

**Table 2:** Candidate drug molecules (top ten) identified from gene-drug interaction enrichment analysis.

Drugs	Adjusted p-value	Combined score	Related genes
Fenofibrate	0.008098833	4973.589533	JUN; ESR1
Cinnarizine	0.008098833	4494.847104	JUN; ESR1
Propanil	0.008098833	4094.008378	JUN; ESR1
Fenthion	0.008392041	3208.634563	JUN; ESR1
Clindamycin	0.008392041	170.2000146	UBE2I; RPL37A; MRPL16; MRPL17; RPL26L1; MRPL22
Chloramphenicol	0.008392041	2463.818516	JUN; ESR1
Demeclocycline Hydrochloride	0.008392041	2199.345383	UBE2I; ESR1
Azacitidine	0.008392041	263.5050207	UBE2I; MRPS11; RPL37A; ESR1
Chrysene	0.008392041	2085.577517	JUN; ESR1
Artenimol	0.008995676	1887.299561	JUN; ESR1

The first column indicated the names of the candidate drug molecules. The second column indicated the adjusted p-value (the p-value adjusted via Benjamini and Hochberg (FDR) of the corresponding drugs; the smaller the value, the more significant the drug). The third column indicated the combined score of each molecule drug. The forth column indicated the correlated genes of each drug.

### Molecular docking analysis

**Fig 7:**
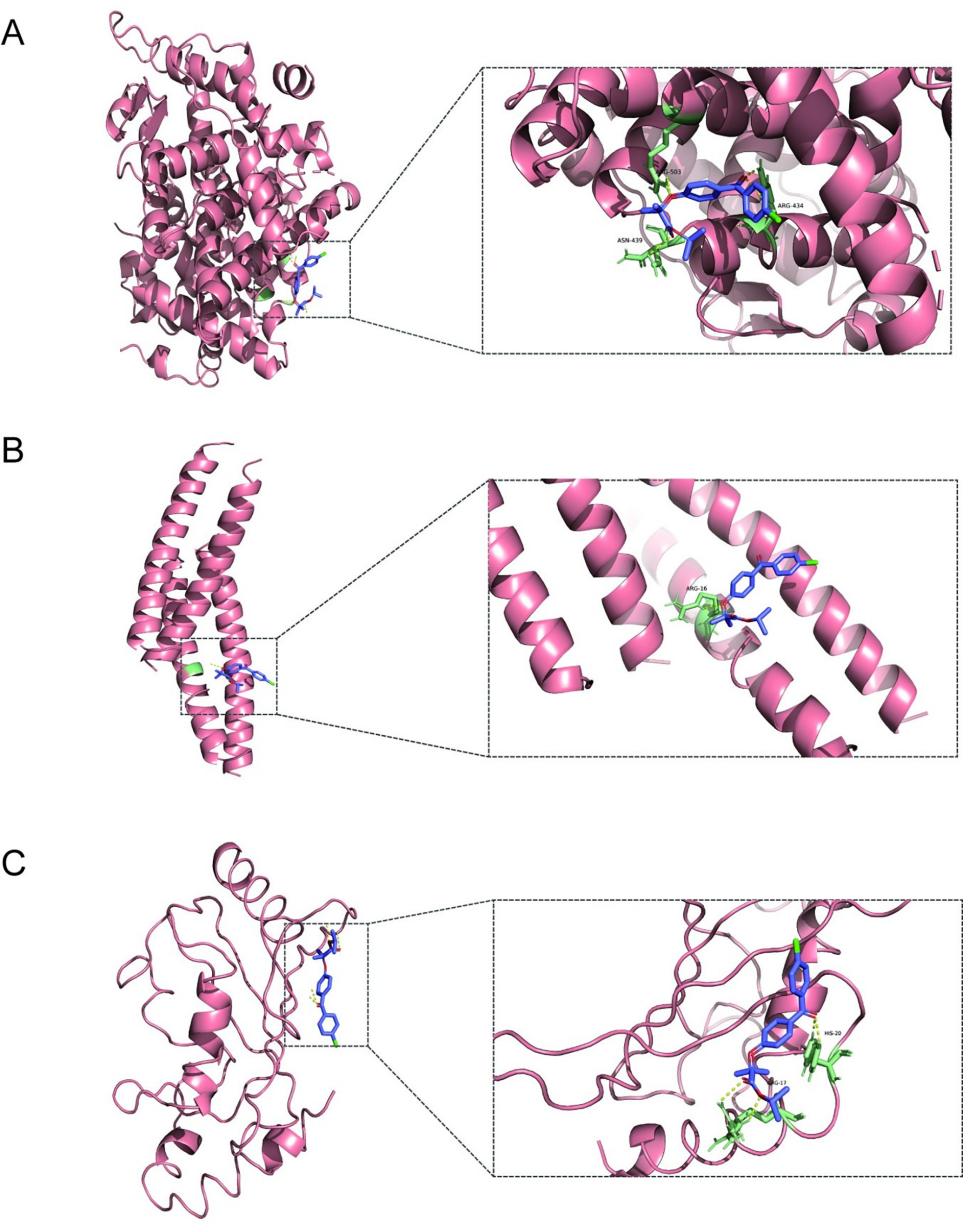
Molecular docking of fenofibrate with proteins of hub genes. (A). The binding poses of ESR1 complexed with fenofibrate. (B). The binding poses of JUN complexed with fenofibrate. (C). The binding poses of UBE2I complexed with fenofibrate. Hydrogen bonds are indicated as dashed lines.

Molecular docking was performed to evaluate the binding affinity of fenofibrate to 10 hub targets. A lower affinity score indicates stronger binding ability. The crystal structures of MRPL16, MRPL17, RPL26L1, MRPL22, RPL37A and GTF2H3 were not available in PDB database, thus molecular docking analysis was only performed for JUN(PDB ID:5FV8), ESR1(PDB ID:3UUD) and UBE2I(PDB ID:5F6E). Docking affinity scores for fenofibrate against JUN, ESR1 and UBE2I were all less than -1.2 kcal/mol implying that these compounds have reasonable binding affinities with the hub proteins (Fig 7 and Table 3).

**Table 3:** Docking parameters and results.

Targets	Box center (x, y, z)	Box size (x×y×z)	Docking affinity (kcal/mol)
JUN	26.45, 13.17, 3.21	40×40×56	-6.0
ESR1	22.76, 4.85, 6.01	68×64×72	-5.4
UBE2I	54.35,0.42, 13.94	44×46×40	-7.2

The first column indicated names of protein targets for molecular docking with fenofibrate. The second column indicated the box center of molecular docking of each target. The third column indicated the box size of molecular docking of each target. The fourth column indicated the docking affinity scores of each target with fenofibrate. The smaller the value, the higher the protein binding affinity.

## Discussion

EC is the most common malignancy type in females in the developed world and is associated with high incidence and mortality rate [25]. Approximately 60,000 females are diagnosed with EC, and 10,000 deaths are recorded each year [26]. Accordingly, in order to prevent it, identifying women at high risk of EC is important. Women with PCOS presenting with a 9% lifetime risk of EC are considered as a high-risk group for EC. Several clinical features of PCOS including obesity, insulin resistance, unregulated estrogen stimulation of the endometrium, diabetes and progesterone resistance are metabolic and molecular risk factors for EC [8]. However, the exact relationship between PCOS and EC has not been fully elucidated.

**Fig 8:**
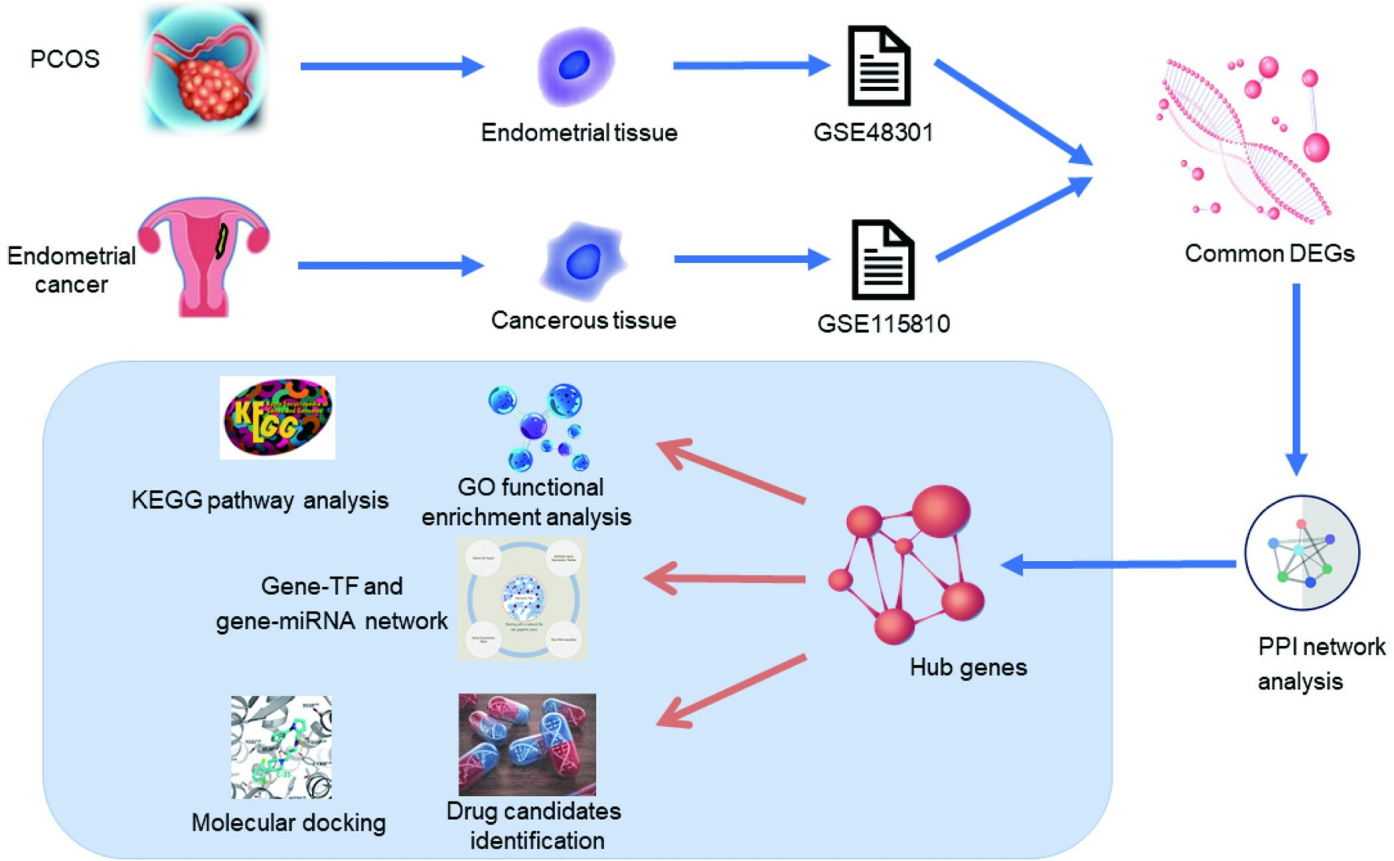
Research flowchart.

**Fig 9:**
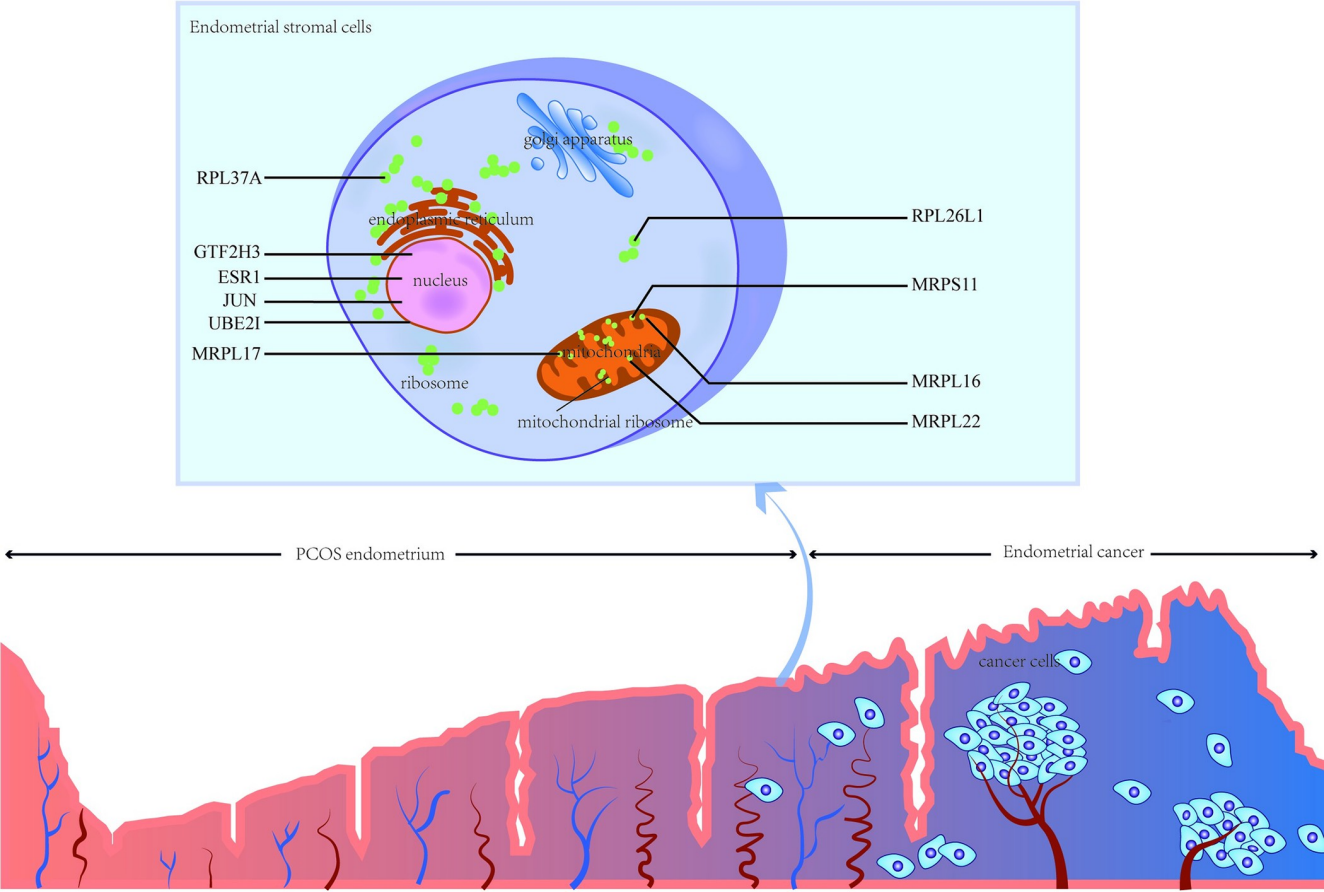
Brief schematic diagram. A speculative mechanism diagram of the hub genes in PCOS endometrium carcinogenesis.

In the present study, bioinformatics analyses were used to identify hub genes for PCOS and EC, and to explore the transcriptional regulatory signatures for these genes. Notably, a total of 10 hub genes namely, MRPL16, MRPL22, MRPS11, RPL26L1, ESR1, JUN, UBE2I, MRPL17, RPL37A and GTF2H3 were identified from the DEGs of PCOS and EC endometrial tissues. GO analysis and KEGG pathway analysis, construction of genes-TFs and genes-miRNAs interaction networks, and small molecule drug prediction were performed to further explore the role of the hub genes. The finding showed that ribosome and mitochondrial translation were the most important common pathways for PCOS and EC, and ten drug molecules led by fenofibrate was detected as potential drugs to decrease EC risk for PCOS patients (Figs 8 and 9).

KEGG pathway enrichment analysis results showed that ribosome associated with MRPL16, MRPL22, MRPS11, RPL26L1 MRPL17, RPL37A plays a key role in pathogenesis of PCOS and EC. Ribosome is a complex organelle involved in mRNA translation and protein synthesis [27]. Abundant findings indicate that hyperactive ribosome biogenesis promotes tumorigenesis through quantitative and qualitative changes in ribosomes that affect the process of translation [28]. Additionally, GO terms related to hub genes reveal that mitochondrial translation is implicated in the cancerous process in endometrial tissue of PCOS. Translation of mRNA involved in transcription of mtDNA into protein in mitochondrial ribosome comprises initiation, elongation and termination steps, which are regulated by several mammalian mitochondrial translation factors such as mtIF2, mtIF3, and mtEF4 [29]. Translation process in mitoribosomes is linked to oxidative phosphorylation (OXPHOS) defects and results in increased oxidant stress [30, 31]. Recent studies report that alteration in the level of mitoribosome translation is implicated in development and progression of tumors [32]. Notably, components of the mitochondrial translational machinery are potential targets for tumor treatment. For instance, inhibition of the mitochondrial translation elongation factor, EF-Tu in acute myeloid leukemia attenuates cell growth and improves tigecycline sensitivity [33]. A previous study using 42 patient biopsies reported that MRPS18-2 was significantly highly expressed in EC compared with the level in normal endometrium [34]. However, no evidence has explored the relationship between mitoribosome translation and PCOS. Further studies are strongly recommended to explore the detailed mechanism of mitoribosome translation in PCOS and EC.

TFs control gene expression by directly binding to DNA sequences of target genes thus play a regulatory role in transcription and translation processes [35, 36]. Moreover, miRNAs modulate mRNA translation and transcript degradation [37]. TFs and miRNAs modulate genetic expressions which may result in formation of cancer cells [38]. The hub genes identified in the current study were uploaded in network analyst platform for analysis of TF-genes interaction networks to identify TF associated with PCOS and EC. Analysis of the network showed that KLF9, PHF8, KDM5B and SAP30 were significantly correlated TFs with hub genes. They have all been shown to take part in tumorigenesis [39–42]. Moreover, previous studies have suggested that KLF9 is a tumor suppressor involved in development of EC [43].

DSigDB database was used to identify potential molecular drugs for the 10 hub genes. The findings showed that fenofibrate was potential drug molecule for the hub genes. Fenofibrate is PPARα agonist and is widely applied in clinical practice as an effective lipid-lowering agent. Fenofibrate exerts its activity by increasing HDL levels and decreasing the levels of LDL, cholesterol and triglycerides [44]. Hyperlipidemia is a common feature in women with PCOS and is associated with several clinical characteristics of PCOS such as IR, hyperandrogenemia, anovulation and inflammation. Although it is not the first-line treatment for lipid-lowering in PCOS, fenofibrate is recommended owing to few drug interactions and low muscle toxicity [45]. Fenofibrate was recently reported to exert anticancer effects in various of human tumors [46–48]. A previous study reported that fenofibrate inhibited proliferation and induced apoptosis in Ishikawa endometrial cancer cells [49]. Moreover, it promotes metabolism of fatty acids over glucose for the metabolic needs in tumor microenvironment thus decreasing tumor progression [50]. In order to explore the therapeutic potential of fenofibrate, we started a preliminary validation through molecular docking, which can predict binding affinities between molecule and protein residues using binding free energy (ΔGbind) [51]. Findings from molecular docking showed that fenofibrate interacted directly with the active residues of the target proteins including JUN, ESR1 and UBE2I via multiple hydrogen bonds. These findings indicate that fenofibrate is a potential candidate for future drug development targeting both EC and PCOS. Future experimental studies are required to test its potential in the treatment of PCOS patients with EC.

## Conclusion

Currently, the relationship between PCOS and EC has not been completely understood. This is the first study to explore the association between PCOS and EC using an omics data based combined approach. Common DEGs were identified by screening genome expression data of different endometrial cells. Gene signatures and regulatory signatures were identified through bioinformatics analysis. Moreover, the molecular mechanisms of these signatures were explored and potential small drug molecules associated with the hub genes were identified. Further experimental and clinical studies should be conducted to verify the identified molecular signatures and potential drugs.

## Supporting information

S1 Table GEO dataset of PCOS patients. (XLS)

S2 Table GEO dataset of endometrial cancer patients. (XLS)

S3 Table PPI network of hub genes. (XLS)

S4 Table Transcription factors (TFs) that regulate hub genes. (XLS)

S5 Table miRNAs that regulate hub genes. (XLS)

S6 Table Potential drug candidates. (XLS)
